# Supplementary material for: Evaluating Families’ Opinions of Routine Influenza Vaccination in Children Under 5 Years of Age in Spain
Source: Vaccines (Basel). 2025 Jan 10;13(1):54. doi: 10.3390/vaccines13010054 (PMC11768964; doi:10.3390/vaccines13010054)
Supplement: Supplementary file 1 [file vaccines-13-00054-s001.zip › vaccines-3367062-Supplementary.pdf]

SUPPLEMENTARY MATERIAL

Supplement S1. Survey

Influenza is a very prevalent infectious disease and has a major impact on public health. This year in Catalonia, influenza vaccination has been included in the routine vaccination schedule for all children under 5 years of age. For this reason, a group of researchers from Central Catalonia is promoting a study on influenza vaccination in paediatrics. We want to know how families feel about the influenza vaccine.

We would appreciate it if you could answer the following survey.

The decision to participate in the survey is voluntary. All data collected are anonymous, and under no circumstances can the research team identify the individuals who completed the survey. Procedures will be strictly followed to ensure the confidentiality of the information and the anonymity of participants across all research materials. As the data is anonymous, signing an informed consent form is not required. However, by completing the survey, participants are considered to have given their consent to participate in the study.

Thank you very much for your participation!

1. Date of birth:

2. Sex:

   ___Male
   ___Female
   ___ Non-binary

3. Country of origin:
   ___Spain
   ___ Rest of Europe
   ___ North Africa
   ___ Rest of Africa
   ___ South America
   ___ Central America
   ___ North America
   ___Asia
   ___Other

4. Educational level:
   ___Primary
   ___Secondary
   ___College
   ___Further Education
   ___ Higher Education

5. Number of children:
   ___ 1
   ___ 2
   ___ 3
   ___ 4
   ___ 5
   ___ 6
   ___ >6

6. Within what age range are each of your children of paediatric age? (You can choose more than one option):

___ 6 months-2 years
___ 3-5 years
___ 6-10 years
___ 11-14 years

7. Do your children have any illnesses?
___Yes
___No

8. What illnesses do your children have? ___________

9. Have you ever vaccinated your child against influenza?

___Yes
___No
___I don't know

10. Why did you vaccinate them?
___ Because they are a child with a risk disease
___ Because there is a person at home with a risk disease
___ Because the paediatrician recommended it to me
___Other

11. What was the reason for not vaccinating them?
___ I am afraid to vaccinate them. I am afraid of the unwanted effects of the vaccine.
___ The influenza vaccine is not effective.
___ Influenza is not a serious illness for children.
___ The paediatric professional has not recommended it to me.
___ I don't have enough information about the vaccine.
___ In general, I do not trust vaccination.
___ I have had a bad experience with influenza vaccination.
___ My child does not have any illness.
___Other

Influenza vaccination 2023/24

Starting this period 2023/24, the influenza vaccine will be included in the routine vaccination schedule for all children under 5 years of age. We would be interested to know your opinion regarding influenza vaccination in minors.

12. If influenza vaccination is indicated, do you intend to vaccinate your child against influenza this year?
___Yes
___No

13. What are your reasons for vaccinating your child against influenza? (You can choose more than one option):
___ To protect him/her from influenza.
___ To protect the general population from influenza and especially the elderly and those with risk diseases.
___ Because the paediatrician has recommended it to me.
___ Because I have had influenza in the past and know what the consequences may be.
___ Because I have enough information about the vaccine and I think it is necessary.
___ Because other relatives or acquaintances have vaccinated their children and I trust their judgement.
___ Other

14. What reasons do you have for not vaccinating your child against the influenza? (You can choose more than one option):
___ The influenza vaccine is not effective.
___ Influenza is not a major illness. It's okay to catch it.
___ The paediatric professional has not recommended it to me.

___ I don't have enough information about the vaccine.
___ In general, I don't trust any vaccine.
___ I have had a bad experience with influenza vaccination.
___ I feel bad that my child would have to be pricked with a needle. Vaccination is painful.
___ I am afraid of the unwanted effects of vaccination.
___ I have no reason not to vaccinate them. I'll vaccinate them.
___ Other

15. Do you agree that everyone under 5 years of age should be vaccinated against influenza?
___Yes
___No
___ I have no opinion

16. Have you ever had an influenza vaccination?
___Yes
___No
___ I don't know

17. Do you get an influenza vaccination every year?
__ Yes
__ No

18. What is the reason for your vaccination? (You can choose more than one option):
___ Because I am a person with a risk disease.
___ Because I am a relative of a person with a risk disease.
___ Because I am a healthcare worker.
___ Other

19. Why don't you get the influenza vaccination every year? (You can choose more than one option):
___ I have only been vaccinated when a professional has recommended it.
___ I have only been vaccinated when I have been in contact with people with a risk disease.
___ I'm not sure how effective the influenza vaccine is.
___ Other
